# Identifying octogenarians with non-small cell lung cancer who could benefit from surgery: A population-based predictive model

**DOI:** 10.3389/fsurg.2022.972014

**Published:** 2022-07-28

**Authors:** Ce Chao, Dongmei Di, Min Wang, Yang Liu, Bin Wang, Yongxiang Qian

**Affiliations:** Department of Cardiothoracic Surgery, The Third Affiliated Hospital of Soochow University, Changzhou, China

**Keywords:** Octogenarians, NSCLC, surgery, SEER, nomogram

## Abstract

**Background:**

As the population ages, there will be an increasing number of octogenarian patients with non-small cell lung cancer (NSCLC). In carefully selected elderly patients, surgery can improve long-term survival. To identify candidates who would benefit from surgery, we performed this study and built a predictive model.

**Materials and methods:**

Data from NSCLC patients over 80 years old were obtained from the Surveillance, Epidemiology and End Results database. A 1:1 propensity score matching was performed to balance the clinicopathological features between the surgery and non-surgery groups. Kaplan-Meier analyses and log-rank tests were used to assess the significance of surgery to outcome, and Cox proportional-hazards regression and competing risk model were conducted to determine the independent prognostic factors for these patients. A nomogram was built using multivariable logistic analyses to predict candidates for surgery based on preoperative factors.

**Results:**

The final study population of 31,462 patients were divided into surgery and non-surgery groups. The median cancer-specific survival time respectively was 53 vs. 13 months. The patients’ age, sex, race, Tumor, Node, Metastasis score, stage, chemotherapy use, tumor histology and nuclear grade were independent prognostic factors. Apart from race and chemotherapy, other variates were included in the predictive model to distinguish the optimal surgical octogenarian candidates with NSCLC. Internal and external validation confirmed the efficacy of this model.

**Conclusion:**

Surgery improved the survival time of octogenarian NSCLC patients. A novel nomogram was built to help clinicians make the decision to perform surgery on elderly patients with NSCLC.

## Introduction

Lung cancer is the leading cause of death in the global cancer population (1, 2). In 2020, lung cancer was the second most common cancer and the leading cause of cancer-related deaths worldwide (2). Non-small cell lung cancer (NSCLC) accounts for about 80% of lung cancers. The average age of patients diagnosed with lung cancer is about 70 years old, and the incidence of lung cancer increases with age (1). In Europe, patients younger than 50 years old account for about 6% of lung cancer patients, while 44% of patients diagnosed with lung cancer are over 70 years old (3). In 2018, there were 2.3 million new cancer cases diagnosed in patients over 80 years old worldwide, accounting for 13.3% of all new global cancer cases (4). With the increase in the elderly population base, the number of new cancer cases rises annually. An estimated 6.9 million new cancer cases are expected to be diagnosed in patients over 80 years old worldwide, representing 21.5% of global cases by 2050 (4). Thoracic surgeons will soon face an increasing number of elderly NSCLC patients eligible for surgical treatment.

The management of elderly NSCLC patients is complicated because of age-related physiological changes, comorbidities, frailty, and shorter relative life expectancies (5). At present, it is lack of evidence for elderly patients with NSCLC to guide appropriate treatment decisions. A higher operative mortality rate has been observed in octogenarians compared with patients 60 to 79 years old (6), however, surgical resection of tumors is justified and is an effective treatment for octogenarian and older patients with early-stage NSCLC (7, 8). Nevertheless, several studies have focused on the differences in outcome between octogenarian patients who have undergone surgery and those who have not. Ganti et al. demonstrated in a cohort of 1,338 elderly NSCLC patients that surgery is an independent factor in stage I and stage II NSCLC octogenarians (9). Surgical resection is also the primary treatment choice for a subset of elderly patients with advanced NSCLC (10). Since patients greater than 80 years old are seldom included in randomized clinical trials, prospective studies that are relevant to elderly NSCLC patients are lacking. However, retrospective analyses suggest that surgery should not be abandoned according to age alone in octogenarians with NSCLC (3, 9, 11). In addition, age is not completely representative of an individual's physiological or functional status and should not be used as the sole determining factor for clinical decision-making (12, 13). In the clinic, the choice of surgery for octogenarians depends more on the experience of clinicians from large medical centers, and is therefore not supported by scientific data.

In this study, we attempt to build a visual nomogram to distinguish the optimal surgical candidates among NSCLC patients 80 years old and over, which provides a reliable method for clinicians to make the decision whether to recommend surgery or not.

## Materials and methods

### Study population

The Surveillance, Epidemiology and End Results (SEER) database was used, and the study population was primarily NSCLC patients at least 80 years old between 2014 and 2018. These data were collected using the SEER*Stat Software (version 8.3.9). The matched variables including patient ID, gender, age at diagnosis, race, tumor site, histology, nuclear grade, tumor size, Tumor, Node, Metastasis (TNM) stage, surgical method, radiotherapy, chemotherapy, overall survival (OS), cancer-specific survival (CSS), and survival months, were acquired from this database. The TNM stage of patients was reclassified according to the American Joint Committee on Cancer eighth edition. All data were from the SEER database and anonymized. It is unnecessary to obtain the ethical approval and consent for participants.

**Figure 1:**
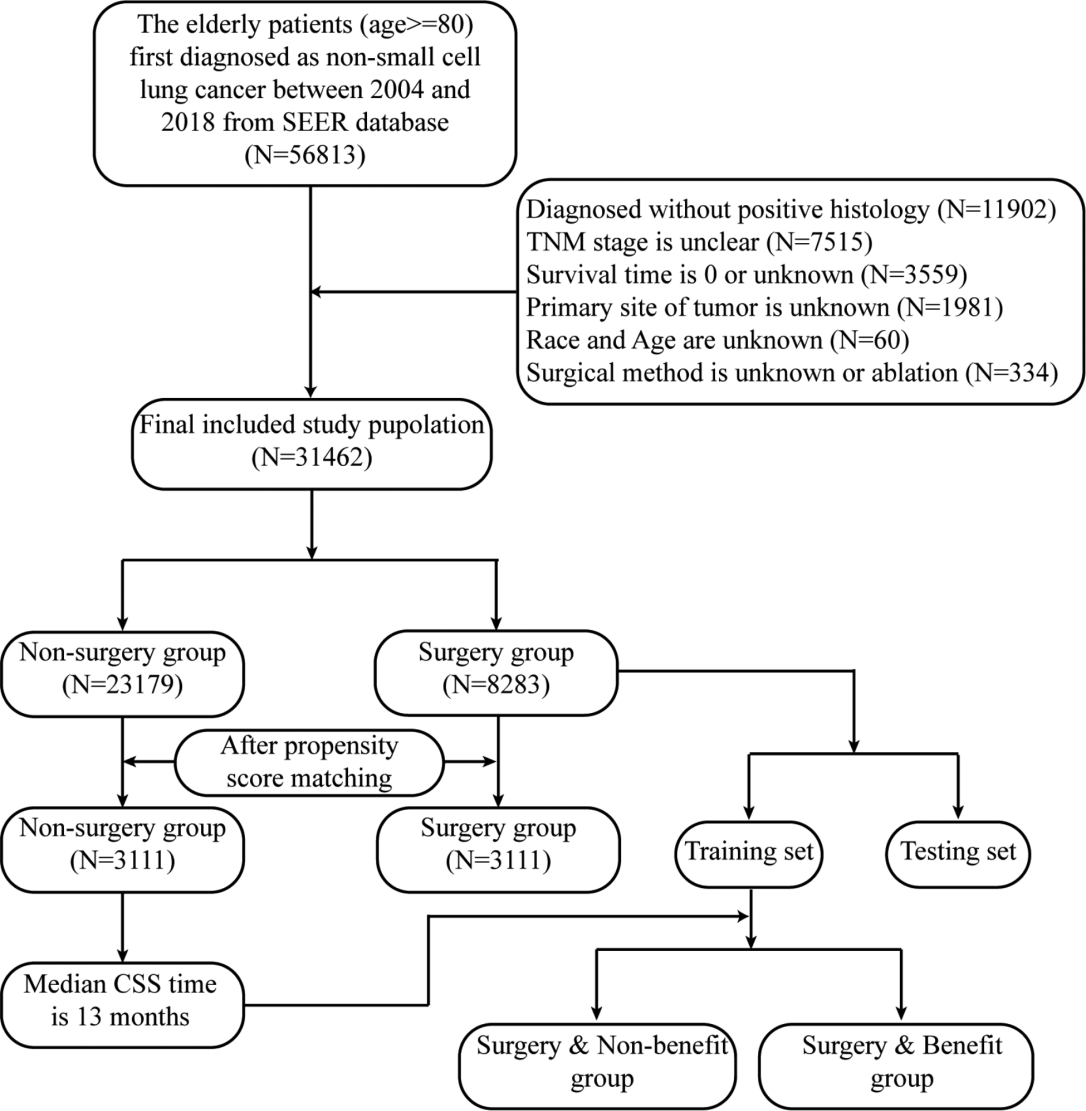
The flowchart of determining final study population.

Based on the following exclusion and inclusion criteria, the final study population was confirmed. The inclusion criteria were as follows: initial diagnosis with primary NSCLC occurring between 2014 and 2018, at least 80 years old, a surgical code of 0, 21–22, and 30–70. The exclusion criteria were as follows: unknown race, age, and tumor site, unclear TNM stage and histology, unknown or null survival time. The detailed flowchart is shown in Figure 1.

### Statistical analyses

A Pearson's chi-squared and Student's t-test were used to compare baseline data and clinical characteristics of patients between the surgery and non-surgery groups, respectively. The Kaplan-Meier method and log-rank test were used to confirm the surgical benefit for the NSCLC patients aged 80 years old and over. A 1:1 matched propensity score matching (PSM) was conducted to balance the variables between the surgery and non-surgery groups using the “MatchIt” R package. Variables including patient age, race, sex, tumor site, nuclear grade, histology type, T stage, N stage, M stage, radiotherapy, and chemotherapy were included in the logistical model to calculate the propensity score with a caliper of 0.01. The univariate and multivariate Cox proportional-hazards regression and competing risk model were performed to identify the independent factors related to the prognosis. R software (version 4.1.2) was used to conduct the statistical analyses with two-sided testing. A *P* < 0.05 was considered statistically significant.

### Nomogram construction and validation

Compared with patients in the non-surgery group, patients undergoing surgery had a longer OS and CSS time. The median CSS of patients in the surgery group was 53 months, while one patient in the non-surgery group had a CSS of 13 months. We assumed that patients who underwent surgery and who had longer CSS times than patients who did not undergo surgery could benefit from a primary tumor resection. Based on this, patients undergoing surgery were randomly divided into a training and testing dataset using a ratio of 7:3.

The “rms” R package and R software (version 4.1.2) were used to complete the construction of the nomogram. Univariate and multivariable logistic analyses were performed to select the final variables included in the predictive model among the variables that had been identified as independent factors for prognosis. The nomogram was built using the multivariable logistic regression analyses based on variables including age, sex, histology type, nuclear grade, T stage, N stage, and M stage.

To test the discriminatory ability and accuracy of this nomogram, internal and external validations were conducted. The ROC curve, calibration plots, and decision curve analysis (DCA) plots were utilized in training and testing datasets, respectively. The predictive model was tested in the population after PSM. The Kaplan-Meier curve was drawn to compare the CSS time of the surgery with benefit group, the surgery without benefit group, and the non-surgery group.

## Results

### Study population pre- and post-PSM

Based on the exclusion and inclusion criteria, the final study population from SEER database included 31,462 patients at least 80 years old and diagnosed with a primary NSCLC. Only 8,283 (26.3%) of octogenarians received surgical treatment. Among them, more than half of patients had a TNM I stage with adenocarcinoma. Surgery was more likely in the white and female population. Patients who did not receive surgical treatment had more access to radiotherapy and chemotherapy. The number of octogenarians with NSCLC have gradually increased in the last decade. However, patients receiving surgery made up a relatively small proportion of this group (Supplementary Figure A1). After PSM, 6,222 patients were included into the matched population and the variables between the surgery and non-surgery groups were balanced (Table 1).

**Table 1:** The clinicopathologic baselines of patients between two groups in elderly patients with NSCLC before and after propensity score matching.

Characteristic	Total patient (*N* = 31,462)	Population before PSM		SMD	Population after PSM		SMD
		Non-surgery group (*N* = 23,179)	Surgery group (*N* = 8,283)		Non-surgery group (*N* = 1,014)	Surgery group (*N* = 1,014)	
Race				0.173			0.072
White	26558 (84.4%)	19244 (83.0%)	7314 (88.3%)		2684 (86.3%)	2608 (83.8%)	
Black	2202 (7.0%)	1854 (8.0%)	348 (4.2%)		179 (5.8%)	196 (6.3%)	
Other	2702 (8.6%)	2081 (9.0%)	621 (7.5%)		248 (8.0%)	307 (9.9%)	
Sex				0.058			0.008
Female	16241 (51.6%)	11790 (50.9%)	4451 (53.7%)		1629 (52.4%)	1617 (52.0%)	
Male	15221 (48.4%)	11389 (49.1%)	3832 (46.3%)		1482 (47.6%)	1494 (48.0%)	
Age (Mean (SD))	83.61 (3.19)	83.93 (3.33)	82.74 (2.56)	0.402	83.34 (2.95%)	83.43 (2.96%)	0.031
Primary Site				0.236			0.072
Upper lobe	17984 (57.2%)	13323 (57.5%)	4661 (56.3%)		1735 (55.8%)	1655 (53.2%)	
Middle lobe	1462 (4.6%)	1005 (4.3%)	457 (5.5%)		148 (4.8%)	175 (5.6%)	
Lower lobe	11007 (35.0%)	7945 (34.3%)	3062 (37.0%)		1176 (37.8%)	1216 (39.1%)	
Main bronchus	692 (2.2%)	679 (2.9%)	13 (0.2%)		16 (0.5%)	13 (0.4%)	
Overlapping lesion	(1.0%)	227 (1.0%)	90 (1.1%)		36 (1.2%)	52 (1.7%)	
Grade				1.131			0.045
I	2782 (8.8%)	1402 (6.0%)	1380 (16.7%)		385 (12.4%)	374 (12.0%)	
II	8131 (25.8%)	4496 (19.4%)	3635 (43.9%)		1048 (33.7%)	1043 (33.5%)	
III	8357 (26.6%)	5735 (24.7%)	2622 (31.7%)		1117 (35.9%)	1119 (36.0%)	
IV	375 (1.2%)	240 (1.0%)	135 (1.6%)		51 (1.6%)	70 (2.3%)	
Unknown	11817 (37.6%)	11306 (48.8%)	511 (6.2%)		510 (16.4%)	505 (16.2%)	
Histology				0.258			0.079
AC	18306 (58.2%)	12895 (55.6%)	5411 (65.3%)		1835 (59.0%)	1862 (59.9%)	
ASC	661 (2.1%)	376 (1.6%)	285 (3.4%)		78 (2.5%)	104 (3.3%)	
LCC	490 (1.6%)	370 (1.6%)	120 (1.4%)		48 (1.5%)	67 (2.2%)	
NEC	614 (2.0%)	498 (2.1%)	116 (1.4%)		63 (2.0%)	66 (2.1%)	
SCC	11391 (36.2%)	9040 (39.0%)	2351 (28.4%)		1087 (34.9%)	1012 (32.5%)	
TNM stage				1.311			0.047
I	9251 (29.4%)	4391 (18.9%)	4860 (58.7%)		1071 (34.4%)	1046 (33.6%)	
II	4119 (13.1%)	2352 (10.1%)	1767 (21.3%)		672 (21.6%)	730 (23.5%)	
III	7730 (24.6%)	6404 (27.6%)	1326 (16.0%)		1021 (32.8%)	1008 (32.4%)	
IV	10362 (32.9%)	10032 (43.3%)	330 (4.0%)		347 (11.2%)	327 (10.5%)	
T stage				0.752			0.053
T1	8398 (26.7%)	5019 (21.7%)	3379 (40.8%)		897 (28.8%)	857 (27.5%)	
T2	8590 (27.3%)	5519 (23.8%)	3071 (37.1%)		991 (31.9%)	991 (31.9%)	
T3	5930 (18.8%)	4841 (20.9%)	1089 (13.1%)		614 (19.7%)	677 (21.8%)	
T4	8544 (27.2%)	7800 (33.7%)	744 (9.0%)		609 (19.6%)	586 (18.8%)	
N stage				0.922			0.112
N0	17544 (55.8%)	10840 (46.8%)	6704 (80.9%)		2128 (68.4%)	2048 (65.8%)	
N1	2714 (8.6%)	1859 (8.0%)	855 (10.3%)		320 (10.3%)	431 (13.9%)	
N2	8746 (27.8%)	8049 (34.7%)	697 (8.4%)		627 (20.2%)	605 (19.4%)	
N3	2458 (7.8%)	2431 (10.5%)	27 (0.3%)		36 (1.2%)	27 (0.9%)	
M stage				1.043			0.021
M0	21100 (67.1%)	13147 (56.7%)	7953 (96.0%)		2764 (88.8%)	2784 (89.5%)	
M1	10362 (32.9%)	10032 (43.3%)	330 (4.0%)		347 (11.2%)	327 (10.5%)	
Radiotherapy				1.142			0.031
Yes	12477 (39.7%)	11938 (51.5%)	539 (6.5%)		503 (16.2%)	539 (17.3%)	
No	18985 (39.7%)	11241 (48.5%)	7744 (93.5%)		2608 (83.8%)	2572 (82.7%)	
Chemotherapy				0.526			0.001
Yes	7794 (24.8%)	6983 (30.1%)	811 (9.8%)		583 (18.7%)	584 (18.8%)	
No	23668 (75.2%)	16196 (69.9%)	7472 (90.2%)		2528 (81.3%)	2527 (81.2%)	

Abbreviations: AC, adenocarcinoma; LCC, large cell carcinoma; NEC, neuroendocrine carcinoma; ASC, adenosquamous carcinoma; SCC, squamous cell carcinoma; PSM, propensity score matching; NSCLC, non-small cell lung cancer; SD, standard deviation; SMD, standard mean difference.

### Surgical benefit for the NSCLC octogenarian group

**Figure 2:**
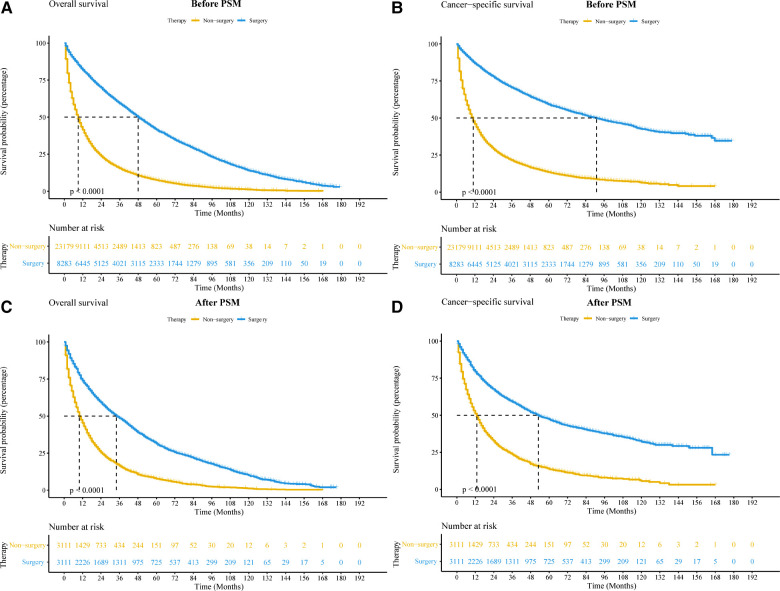
Kaplan-Meier curves of elderly patients with non-small cell lung cancer between surgery and non-surgery groups before and after PSM. (**A,B**) Kaplan-Meier curve comparing OS (**A**) and CSS (**B**) before PSM. (**C,D**): Kaplan-Meier curve comparing OS (**C**) and CSS (**D**) after PSM. Abbreviations: CSS, cancer-special survival; OS, overall survival; PSM, propensity score matching.

As shown in the survival curve, patients who received surgical treatment had longer OS and CSS than patients who did not receive surgical treatment (Figures 2A,B). After PSM, the results of the survival analyses were largely unchanged (Figures 2C,D). The median OS and CSS of the surgery and non-surgery groups were 34 vs. 10 months, and 53 vs. 13 months, respectively. Moreover, the 1-year, 3-year, and 5-year OS and CSS rates are shown in Supplementary Table A1. In the Cumulative Incidence Function, the incidence rate of death from other causes was higher in the surgery group compared with the non-surgery group. However, early death induced by other causes was not significantly different between the surgery and non-surgery groups (Supplementary Figures A2A,B).

### Identification of independent prognostic factors of OS and CSS

A univariate and multivariate Cox proportional-hazards regression and competing risk model were used to identify independent prognostic factors for OS and CSS. The results showed that surgery significantly improved the prognosis of elderly NSCLC patients (OS: HR = 0.37, 95% CI: 0.35–0.39, *P* < 0.001; CSS: HR = 0.40, 95% CI: 0.38–0.43, *P* < 0.001). Age, race, sex, histology type, nuclear grade, T stage, N stage, M stage, and chemotherapy were also independent prognostic factors (Table 2). Although radiotherapy was not a prognostic factor in the multivariate Cox and competing risk model, it was still included in the multivariate analyses to take into account the widespread usage of radiotherapy in elderly NSCLC patients. Radiotherapy could be an independent factor for overall survival. Interestingly, chemotherapy was a protective factor in the multivariate competing risk model, but an unfavorable factor in the univariate competing risk model. In the subgroup analysis, surgery may not improve long-term survival in patients with main bronchus tumor (OS: HR = 0.42, 95% CI: 0.17–1.01, *P* = 0.051; CSS: HR = 0.60, 95% CI: 0.29–1.23, *P* = 0.163). Likely, octogenarians who received radiotherapy did not have a longer cancer-specific survival undergoing surgery (CSS: HR = 0.94, 95% CI: 0.82–1.08, *P* = 0.388). In addition to these, surgery could improve long-term survival in other subgroups (Supplementary Figures A3, A4).

**Table 2:** Univariate and multivariate Cox proportional Hazard analyses and competing risk model for the overall survival and cancer-specific survival in patients with NSCLC after propensity score matching.

Characteristic	Univariate analysis		Multivariate analysis		Univariate analysis		Multivariate analysis	
	Hazard ratio (95% confidence interval)	*P*-value	Hazard ratio (95% confidence interval)	*P*-value	Hazard ratio (95% confidence interval)	*P*-value	Hazard ratio (95% confidence interval)	*P*-value
Surgery								
No	Reference		Reference		Reference		Reference	
Yes	0.41 [0.39, 0.44]	**<0** **.** **001**	0.37 [0.35, 0.39]	**<0**.**001**	0.44 [0.41, 0.46]	**<0**.**001**	0.40 [0.38, 0.43]	**<0**.**001**
Age group								
80–84 years	Reference		Reference		Reference		Reference	
85–89 years	1.07 [1.00, 1.13]	**0**.**043**	1.16 [1.09, 1.23]	**<0**.**001**	0.96 [0.90, 1.03]	0.256	1.08 [1.02, 1.15]	**0**.**014**
90+ years	1.05 [0.91, 1.22]	0.521	1.23 [1.06, 1.43]	**0**.**006**	0.74 [0.62, 0.87]	**<0**.**001**	0.86 [0.73, 1.02]	0.084
Race								
Black	Reference		Reference		Reference		Reference	
White	1.06 [0.95, 1.20]	0.297	0.98 [0.87, 1.10]	0.704	1.05 [0.93, 1.18]	0.435	0.93 [0.83, 1.05]	0.250
Other	0.84 [0.72, 0.97]	**0**.**018**	0.82 [0.71, 0.96]	**0**.**011**	0.84 [0.72, 0.98]	**0**.**029**	0.79 [0.68, 0.92]	**0**.**002**
Gender								
Female	Reference		Reference		Reference		Reference	
Male	1.32 [1.25, 1.40]	**<0**.**001**	1.29 [1.22, 1.37]	**<0**.**001**	1.16 [1.10, 1.23]	**<0**.**001**	1.14 [1.07, 1.20]	**<0**.**001**
Primary site								
Lower lobe	Reference		Reference		Reference		Reference	
Upper lobe	0.92 [0.87, 0.97]	**0**.**004**	0.95 [0.90, 1.01]	0.091	0.97 [0.92, 1.03]	0.353	1.02 [0.96, 1.08]	0.540
Middle lobe	0.88 [0.77, 1.00]	0.056	1.00 [0.87, 1.13]	0.950	0.93 [0.81, 1.06]	0.280	1.04 [0.91, 1.18]	0.554
Main bronchus	1.18 [0.80, 1.76]	0.401	1.07 [0.72, 1.59]	0.743	1.66 [1.18, 2.34]	**0**.**004**	1.31 [0.95, 1.81]	0.099
Overlapping lesion	1.09 [0.87, 1.36]	0.446	1.14 [0.91, 1.43]	0.244	1.27 [1.02, 1.57]	**0**.**031**	1.14 [0.92, 1.41]	0.238
Histology								
AC	Reference		Reference		Reference		Reference	
ASC	1.25 [1.06, 1.47]	**0**.**007**	1.21 [1.03, 1.42]	**0**.**022**	1.19 [1.01, 1.41]	**0**.**039**	1.16 [0.99, 1.37]	0.071
LCC	1.26 [1.04, 1.53]	**0**.**017**	1.21 [0.97, 1.50]	0.088	1.15 [0.94, 1.40]	0.170	1.16 [0.94, 1.43]	0.162
NEC	1.03 [0.84, 1.25]	0.801	0.93 [0.76, 1.14]	0.500	1.08 [0.86, 1.34]	0.517	1.01 [0.79, 1.28]	0.954
SCC	1.28 [1.21, 1.36]	**<0**.**001**	1.19 [1.12, 1.27]	**<0**.**001**	1.15 [1.08, 1.22]	**<0**.**001**	1.07 [1.00, 1.14]	**0**.**046**
Grade		** **		** **		** **		** **
I	Reference	** **	Reference	** **	Reference	** **	Reference	** **
II	1.44 [1.31, 1.58]	**<0**.**001**	1.33 [1.21, 1.46]	**<0**.**001**	1.40 [1.26, 1.55]	**<0**.**001**	1.27 [1.15, 1.41]	**<0**.**001**
III	1.59 [1.45, 1.75]	**<0**.**001**	1.43 [1.30, 1.58]	**<0**.**001**	1.65 [1.49, 1.82]	**<0**.**001**	1.43 [1.29, 1.59]	**<0**.**001**
IV	1.76 [1.44, 2.16]	**<0**.**001**	1.70 [1.35, 2.15]	**<0**.**001**	1.65 [1.34, 2.03]	**<0**.**001**	1.48 [1.15, 1.89]	**0**.**002**
Unknown	1.11 [1.00, 1.24]	0.053	1.19 [1.07, 1.33]	**0**.**002**	1.14 [1.01, 1.29]	**0**.**028**	1.19 [1.06, 1.33]	**0**.**004**
T stage				** **		** **		** **
T1	Reference		Reference	** **	Reference	** **	Reference	** **
T2	1.29 [1.20, 1.39]	**<0**.**001**	1.29 [1.20, 1.39]	**<0**.**001**	1.50 [1.39, 1.62]	**<0**.**001**	1.44 [1.34, 1.56]	**<0**.**001**
T3	1.55 [1.43, 1.68]	**<0**.**001**	1.58 [1.45, 1.71]	**<0**.**001**	1.83 [1.68, 1.99]	**<0**.**001**	1.78 [1.63, 1.93]	**<0**.**001**
T4	1.90 [1.75, 2.06]	**<0**.**001**	1.90 [1.75, 2.07]	**<0**.**001**	2.39 [2.19, 2.60]	**<0**.**001**	2.20 [2.02, 2.40]	**<0**.**001**
N stage		** **		** **		** **		** **
N0	Reference	** **	Reference	** **	Reference	** **	Reference	** **
N1	1.28 [1.17, 1.39]	**<0**.**001**	1.26 [1.16, 1.38]	**<0**.**001**	1.49 [1.37, 1.62]	**<0**.**001**	1.41 [1.29, 1.53]	**<0**.**001**
N2	1.47 [1.37, 1.58]	**<0**.**001**	1.48 [1.38, 1.59]	**<0**.**001**	1.69 [1.58, 1.80]	**<0**.**001**	1.53 [1.42, 1.64]	**<0**.**001**
N3	2.09 [1.60, 2.73]	**<0**.**001**	1.92 [1.46, 2.51]	**<0**.**001**	2.62 [2.09, 3.28]	**<0**.**001**	2.10 [1.66, 2.66]	**<0**.**001**
M stage		** **		** **		** **		** **
M0	Reference	** **	Reference	** **	Reference	** **	Reference	** **
M1	1.86 [1.71, 2.02]	**<0**.**001**	1.78 [1.63, 1.94]	**<0**.**001**	2.12 [1.95, 2.30]	**<0**.**001**	1.82 [1.67, 1.99]	**<0**.**001**
Radiotherapy								
No	Reference		Reference		Reference		Reference	
Yes	0.94 [0.87, 1.01]	0.095	0.86 [0.80, 0.93]	**<0**.**001**	1.01 [0.94, 1.09]	0.717	0.96 [0.89, 1.04]	0.301
Chemotherapy				** **				
No	Reference		Reference	** **	Reference		Reference	
Yes	1.00 [0.94, 1.08]	0.895	0.74 [0.69, 0.80]	**<0**.**001**	1.24 [1.16, 1.32]	**<0**.**001**	0.88 [0.82, 0.95]	**0**.**001**

Abbreviations: AC, adenocarcinoma; LCC, large cell carcinoma; NEC, neuroendocrine carcinoma; ASC, adenosquamous carcinoma; SCC, squamous cell carcinoma; PSM, propensity score matching; NSCLC, non-small cell lung cancer.

### Building the nomogram to identify surgical candidates

**Figure 3:**
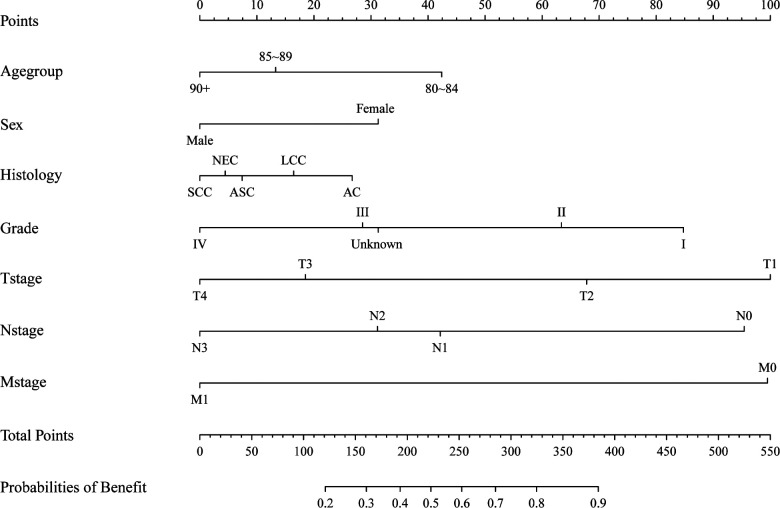
A nomogram to determining surgical beneficial population in elderly patients with non-small cell lung cancer.

We divided the patients in the surgery group into two groups based on their CSS time: surgery with benefit (CSS time >13 months) and surgery without benefit (CSS time ≤13 months). Patients who were alive or died of other cause but did not have more than 13 months of follow-up were excluded. In the end, 7,303 patients were included in the dataset, and 6,216 patients were included in the surgery with benefit group. The whole dataset population was randomly divided into a training and testing dataset using a ratio of 7:3. In the training dataset, univariate and multivariate logistic regression analyses were conducted to identify risk factors of surgical benefit. Variables including age, sex, histology type, nuclear grade, and TNM stage were included in this logistic model. Based on the logistic model, a novel nomogram was drawn using the “rms” R package (Figure 3). In this nomogram, every variable corresponds to a score point.

### Internal and external nomogram validation

**Figure 4:**
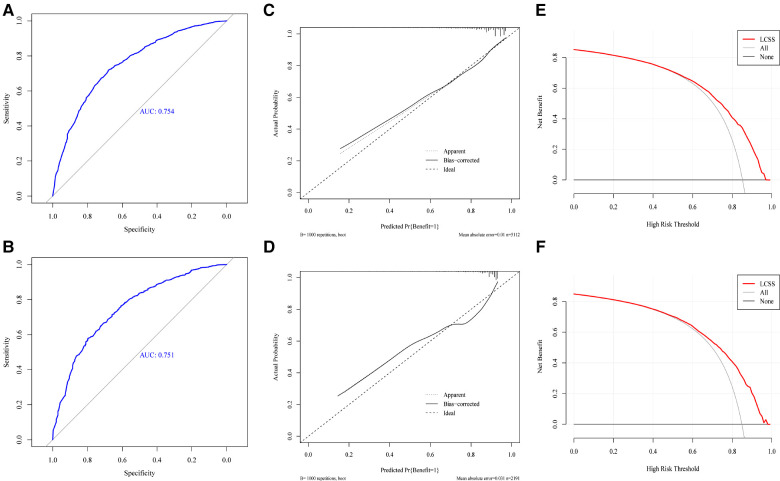
The ROC curve (**A,B**), calibration plots (**C,D**), decision curve analysis curve (**E,F**) of nomogram in training and testing set.

First, we generated an ROC curve, calibration plots, and a DCA curve to measure the discrimination and accuracy of the nomogram in the training set. Subsequently, the same method was used in the testing set. The ROC curves showed that the nomogram had an evident classification effect in the training dataset (AUC: 0.754 (95% CI: 0.736–0.773), Figure 4A) and testing dataset (AUC: 0.751 (95% CI: 0.723–0.780), Figure 4B). The nomogram fit well in the training and testing datasets (Hosmer-lemeshow test: *P* = 0.303 and *P* = 0.367, respectively). The predictive probability of the nomogram was well correlated with the actual probability in the calibration plots (Figures 4C,D). The DCA curves suggested that the nomogram could be applied in clinical decision-making to identify surgical candidates (Figures 4E,F).

**Figure 5:**
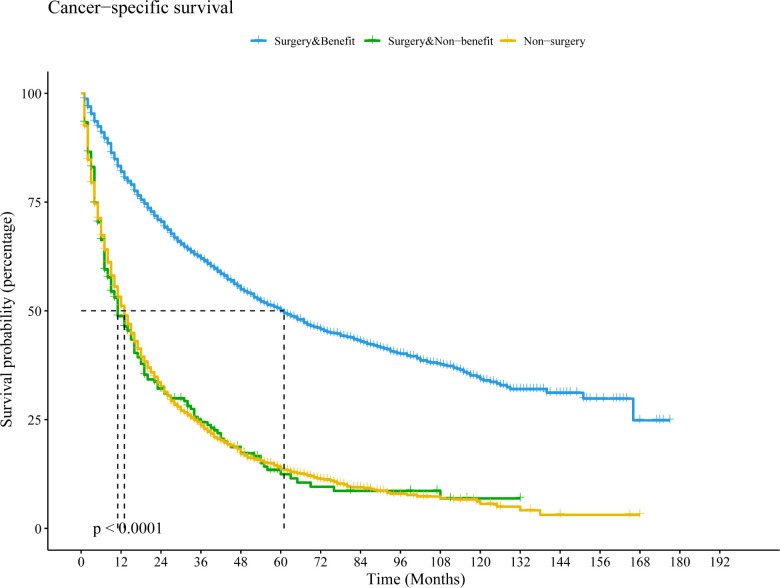
Kaplan–Meier curve to compare differential beneficial groups in the population after PSM based on this nomogram. Abbreviations: PSM, propensity score matching; LCSS, lung cancer-special survival.

We then redivided the population after PSM to test the classification effect of this nomogram. Patients in the surgery with benefit group had a longer CSS time than the surgery without benefit group (HR = 3.08, 95% CI: 2.64–3.60, *P* < 0.001) and the non-surgery group (HR = 3.06, 95% CI: 2.85–3.28, *P* < 0.001). However, there was no significant difference between the surgery without benefit group and the non-surgery group (HR = 0.99, 95% CI: 0.85–1.15, *P* = 0.923) (Figure 5).

## Discussion

As the global population ages, the proportion of octogenarians with NSCLC is increasing annually. Although the health of elderly patients has seen increased attention in the last decade, there is a lack of evidence-based medical treatment approaches to apply to NSCLC patients greater than 80 years old. In recent studies, an increasing number of clinicians have expressed that age is not a limiting factor for surgical treatment for elderly NSCLC patients (5, 14). In this study, we demonstrated that octogenarians with NSCLC could benefit from primary tumor resection. Herein, a novel nomogram was developed to help clinicians identify potential surgical candidates from elderly NSCLC patients. We have shown that this model is effective and stable through ROC curve analyses, calibration plots, DCA curve, and application to the whole cohort.

Previously, thoracic surgeons seldomly performed surgery on octogenarians with NSCLC because of higher postoperative cardiac and pulmonary complications (15). With the development and widespread usage of minimally invasive technology and robotic surgery, the quality of life among octogenarians after surgery remains similar to younger patients (16, 17). Age may not influence the development of complications (18, 19). In our study, age was still an independent factor for prognosis, and surgery did not increase the rate of early death from other causes for octogenarians with NSCLC. Therefore, age only is an important factor for surgery. Elderly males had a poor prognosis, which could be associated with smoking and postoperative complications (20). Lung squamous cell carcinoma was identified as a poor prognostic factor with the univariate and multivariate analysis, which was consistent with previous studies (21). However, nuclear grade, TNM stage, and chemotherapy were also independent prognostic factors and T stage and N stage could potentially be positively associated with prognosis in octogenarians with NSCLC. It is necessary for octogenarians to complete a preoperative examination, especially regarding pulmonary puncture. For OS, radiotherapy was a protective factor. However, the multivariable competing risk model did not support this. It is possible that radiotherapy resulted in more deaths from other causes in elderly patients. A prospective study including multiple centers would be needed to verify this.

In our predictive model, tumor characteristics including differentiation and TNM stage were the factors most related to surgical benefit. The actual TNM stage and pathological information were critical factors for octogenarians with NSCLC. Moreover, younger female octogenarians, or patients with lung adenocarcinoma, could benefit from surgical section. These data suggest that certain patient characteristics could decrease postoperative complications.

There is presently a lack of high-level randomized controlled clinical trials for elderly patients with NSCLC. Octogenarians are usually excluded from randomized controlled clinical trials. In the literature, it has been shown that surgical treatment is feasible for octogenarians with NSCLC, and there are comparable survival rates between octogenarians and younger age groups who underwent surgical resection (8, 9, 17, 22, 23). In addition, minimally invasive surgical resection can improve long-term survival compared with traditional thoracotomy and stereotactic body radiotherapy in early-stage NSCLC (14, 24). Lymph node dissection could limitedly improve long-term survival, but this approach is conducive to subsequent true nodal staging and adjuvant treatment (14, 25, 26). Therefore, wedge or partial resections could be an alternative treatment for octogenarians (27). For advanced NSCLC octogenarians, our model suggests that specific patients may also benefit from surgical resection. However, retrospective and prospective studies in the literature have only focused on the efficiency of chemotherapy and targeted therapy for octogenarians (28–30). In the management of elderly patients (≥65 years old) with NSCLC, surgery is still the main choice for elderly patients (5). In general, treatment-related toxicity increases according with age (10). Patients over 70 years old have similar survival rates compared with patients between 55 and 69 years old with locally advanced NSCLC (31). Therefore, treatment of elderly advanced NSCLC patients should include chemotherapy, radiotherapy, and surgery in select cases (10). In recent studies, stereotactic ablative radiotherapy has emerged as a treatment alternative instead of surgery for early stage NSCLC (32, 33). However, lobectomy offers better survival than stereotactic ablative radiotherapy for patients aged 80 years or more with stage I NSCLC (14). Meanwhile, immunotherapy and targeted therapy are the vital treatment methods for patients with advanced NSCLC. Compared with younger patients, Geriatric patients are more susceptible to developing more serious adverse reactions (34). Even so, epidermal growth factor receptor tyrosine kinase inhibitors (EGFR-TKI) may play an important role for octogenarian patients with NSCLC in the initial treatment modalities (35). And, osimertinib, targeting EGFR T790M-mutated, has been approved in patients with resistance of EGFR-TKI and showed a good therapeutic effect for octogenarian patients with NSCLC (29). Although targeted therapies are expected to be a first-line treatment for advanced NSCLC, it is not ignored to reduce the local tumor burden by surgery. Maybe, comprehensive personalized treatment is the best methods for management of octogenarian patients with NSCLC. Surgery should be performed in carefully selected octogenarian patients with advanced NSCLC.

There are several limitations to our study. First, performance status, comorbidities, and pulmonary function were unknown because of the lack of relevant data in the SEER database. These variables could result in surgical intolerance, serious postoperative complications, and even early death. Secondly, some cancer biomarkers were unknown, such as neuron-specific enolase, carbohydrate antigen 125 and squamous cell carcinoma antigen. Moreover, it has been reported that the C-reactive protein to albumin ratio is related to the prognosis of elderly patients (≥80 years old) (36). However, these serum biomarkers were not collected in the SEER database. Thirdly, neoadjuvant chemoradiotherapy use, the sequence of surgery and chemotherapy, and the method and doses of chemoradiotherapy were unknown in the SEER database.

## Conclusions

Primary tumor resection could improve the long-term survival of octogenarians with NSCLC, and detailed TNM stage and pathological information are necessary prior to treatment. We built a novel nomogram with high specificity and stability to identify octogenarians with NSCLC who could benefit from surgery.

## Data Availability

Publicly available datasets were analyzed in this study. This data can be found here: https://seer.cancer.gov/.

## Supplementary Material

Supplementary Figure 1 The trend of surgery over time in elderly patients with non-small cell lung cancer between 2004 and 2018 year.

Supplementary Figure 2 The cumulative incidence rate plots of elderly patients with non-small cell lung cancer before and after PSM.

Supplementary Figure 3 The forest plots comparing the effect of surgery on overall survival based on different subgroup variables.

Supplementary Figure 4 The forest plots comparing the effect of surgery on cancer-special survival based on different subgroup variables.
