# Supplementary figures and images for: Identifying octogenarians with non-small cell lung cancer who could benefit from surgery: A population-based predictive model
Source: Front Surg. 2022 Jul 28;9:972014. doi: 10.3389/fsurg.2022.972014 (PMC9366359; doi:10.3389/fsurg.2022.972014)

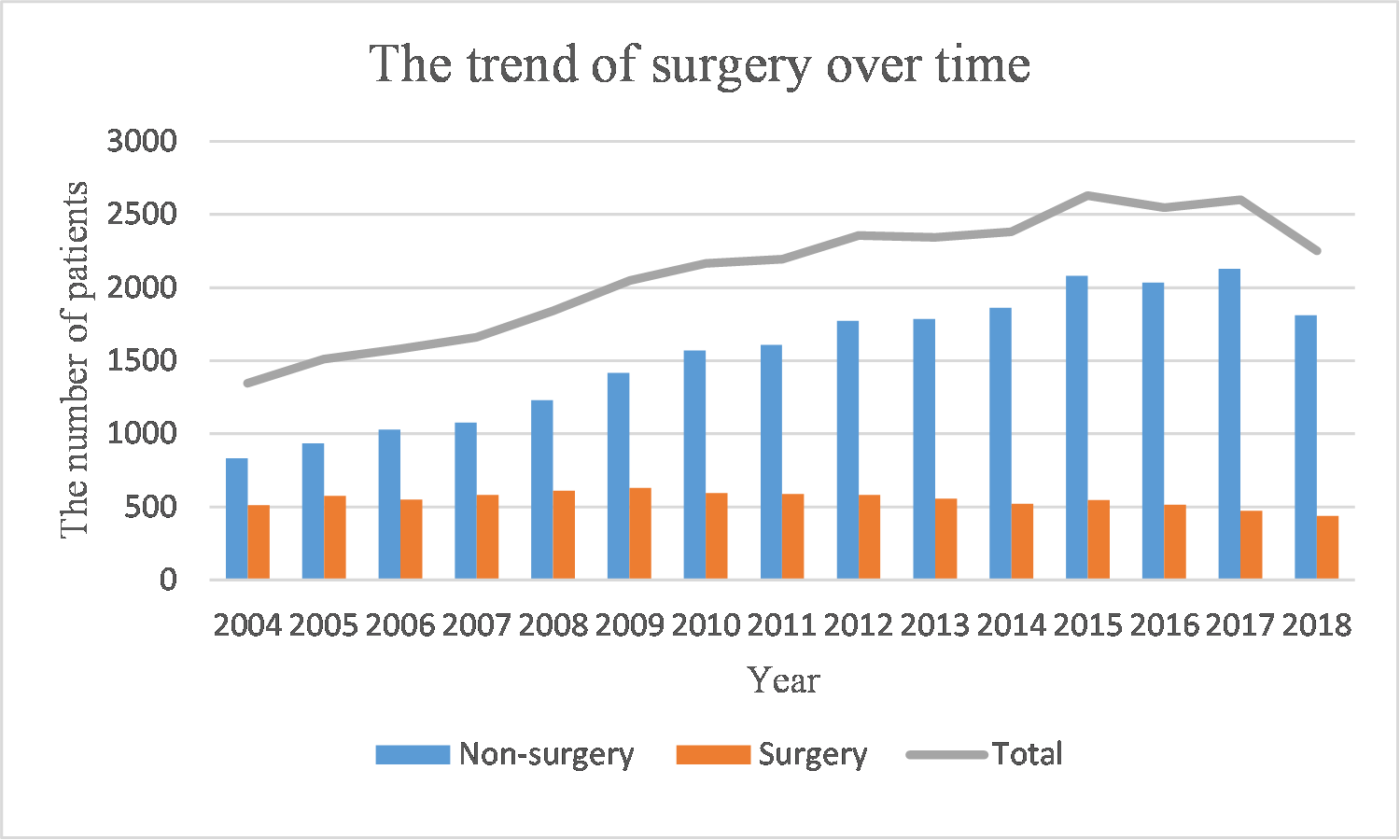

Supplement: Supplementary Figure 1 — The trend of surgery over time in elderly patients with non-small cell lung cancer between 2004 and 2018 year. [file Image_1_v1.tif]

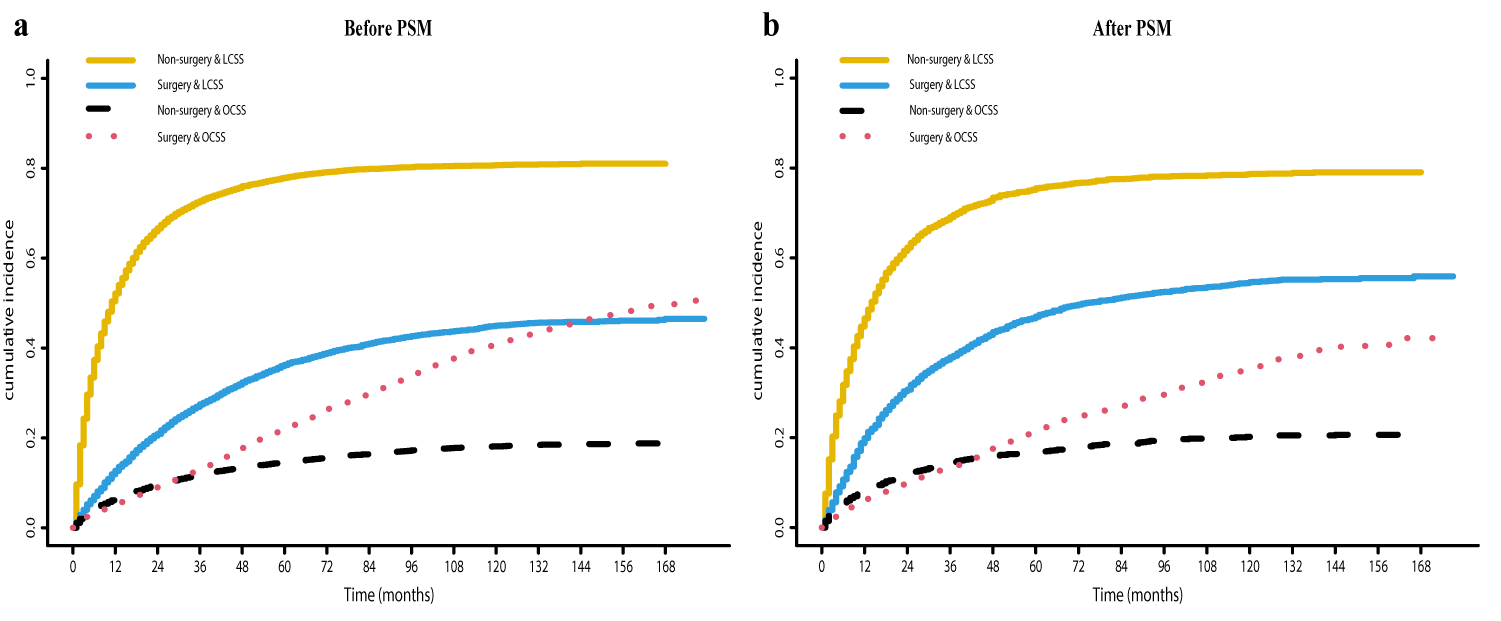

Supplement: Supplementary Figure 2 — The cumulative incidence rate plots of elderly patients with non-small cell lung cancer before and after PSM. [file Image_2_v1.tif]

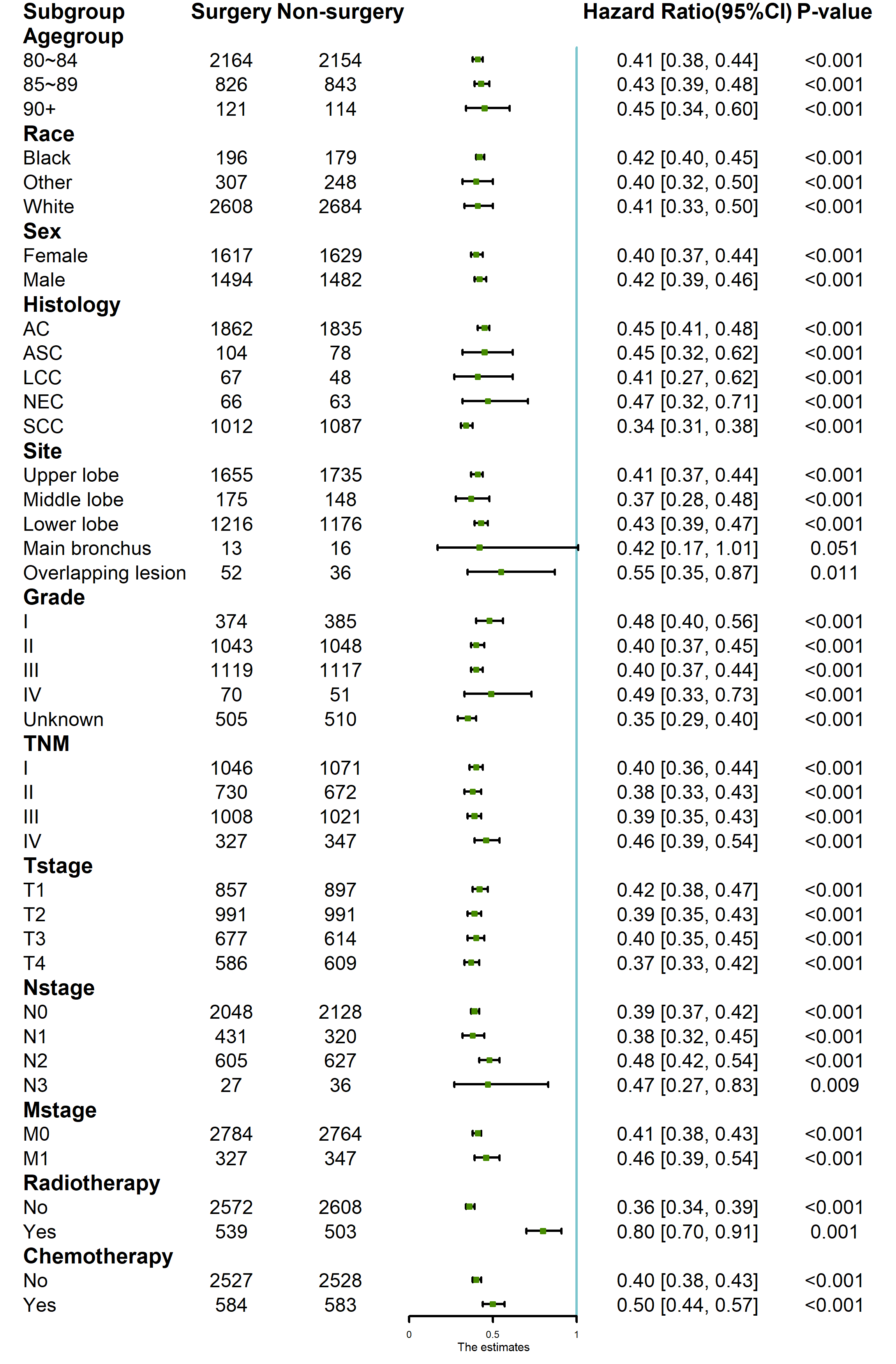

Supplement: Supplementary Figure 3 — The forest plots comparing the effect of surgery on overall survival based on different subgroup variables. [file Image_3_v1.tif]

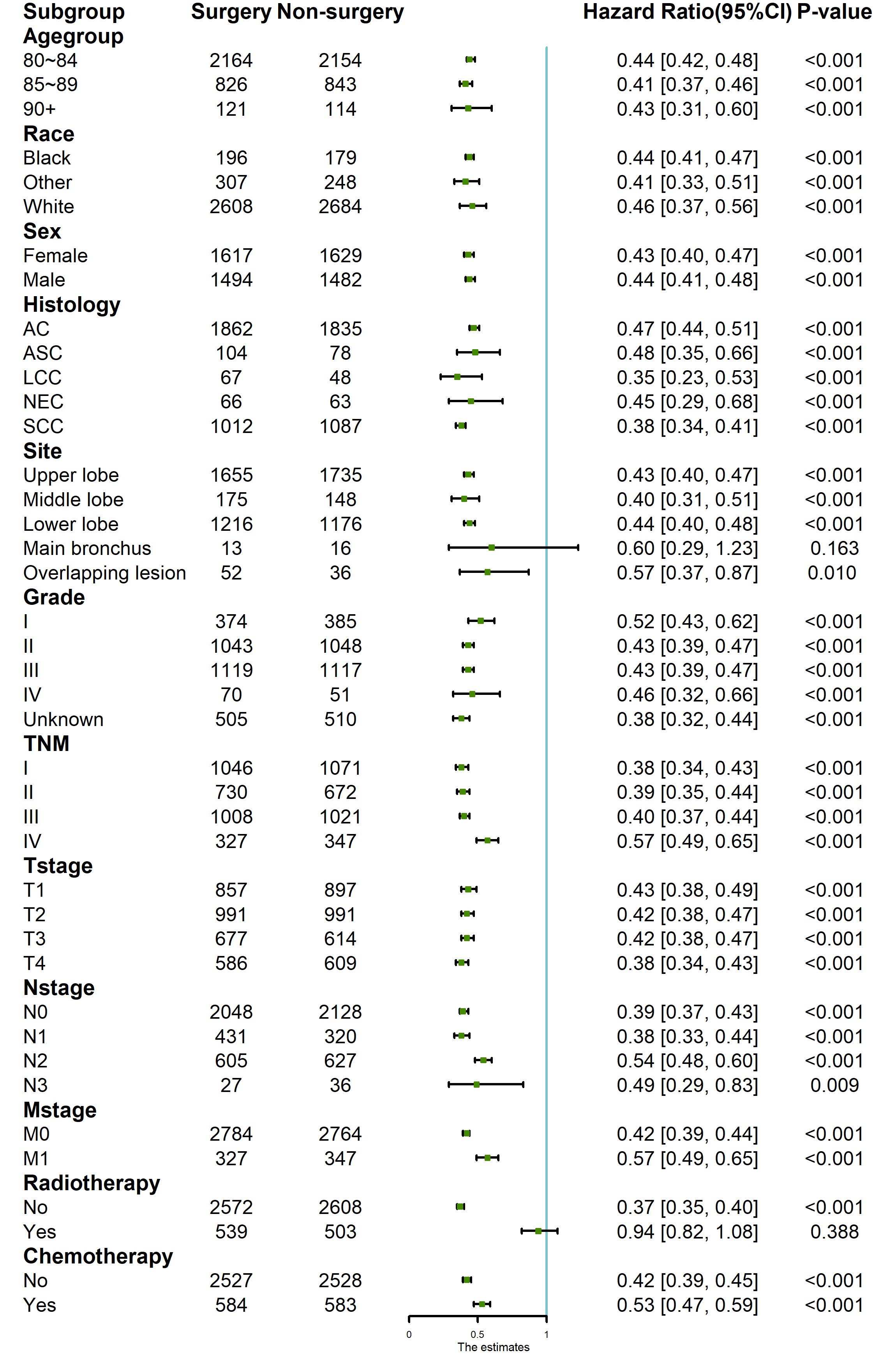

Supplement: Supplementary Figure 4 — The forest plots comparing the effect of surgery on cancer-special survival based on different subgroup variables. [file Image_4_v1.tif]
