# Supplementary material for: Identifying octogenarians with non-small cell lung cancer who could benefit from surgery: A population-based predictive model
Source: Front Surg. 2022 Jul 28;9:972014. doi: 10.3389/fsurg.2022.972014 (PMC9366359; doi:10.3389/fsurg.2022.972014)
Supplement: Supplementary file 5 [file Table_1_v1.docx]

Table S1. The 1-, 3-, 5-year survival rate of overall survival and cancer-specific survival in population after propensity score matching

|  | 1-year | 3-year | 5-year |
| --- | --- | --- | --- |
| overall survival rate |  |  |  |
| Non-surgery group | 45.40% | 16.50% | 7.75% |
| Surgery group | 73.90% | 48.60% | 31.50% |
| cancer-specific survival rate |  |  |  |
| Non-surgery group | 51.10% | 23.60% | 13.60% |
| Surgery group | 79.40% | 59.10% | 47.30% |
